# Evaluation of the characteristics of an AISI 1045 steel quenched in different concentration of polymer solutions of polyvinylpyrrolidone

**DOI:** 10.1038/s41598-020-79060-0

**Published:** 2021-01-14

**Authors:** Eduardo da Rosa Vieira, Luciano Volcanoglo Biehl, Jorge Luis Braz Medeiros, Vagner Machado Costa, Rodrigo Jorge Macedo

**Affiliations:** 1 Programa de Pós-Graduação Em Engenharia Mecânica, Universidade Federal Do Rio Grande Do Sul, Porto Alegre, 90050-170 Brasil; 2 Escola de Engenharia, Fundação Universidade Federal Do Rio Grande, 96203-900 Rio Grande, Brasil; 3 Instituto Federal de Educação, Ciência e Tecnologia Do Rio Grande Do Sul, IFRS, 96201-460 Rio Grande, Brasil

**Keywords:** Mechanical engineering, Mechanical properties, Metals and alloys

## Abstract

Quench hardening aims at the microstructural transformation of steels in order to improve hardness and mechanical strength. The aim phase is, in most cases, the martensite. It is necessary to heat the material until it obtains its austenitization and quenching by immersion in a fluid. Currently, it is common to use watery polymeric solutions in this procedure. These fluids, which are the mixture of polymers in water, vary their thermal exchange capacity depending on the concentrations applied. The increase in concentration minimizes the removal of heat from the part, reducing the formation capacity of martensite, and developing a lower hardness and strong steel. In this work, microstructural characteristics and properties of AISI 1045 steel quenched in solutions based on polyvinylpyrrolidone (PVP) in 10, 15, 20, and 25% concentration were evaluated. The microstructural characterization quantified the percentage of the phases in each concentration, demonstrating a reduction of martensite as the concentrations were high. The investigation of the samples by x-ray diffraction confirmed the absence of austenite retained in the material. Furthermore, a microhardness scale between the core and the surface was constructed, in which a reduction gradient of the indices of this property towards the core of the sample was evidenced.

## Introduction

The purpose of quenching heat treatment is to increase the strength and mechanical resistance of materials through their microstructural transformation. Typically, the microstructure to be obtained is the martensite that forms from the austenite. To obtain the martensite, it is necessary that the material is at a high enough temperature for its austenitization to occur and then be immersed in a fluid at room temperature for its cooling to occur before the carbon diffusion begins^1,2^. Because of the contact of the part at high temperature with the fluid at room temperature, the fluid is vaporized and consecutively develops the steam film, boiling, and convection stages. During the vapor film stage, a vapor fluid forms around the capsule body, preventing heat transfer by conduction and convection, causing a slower cooling, which impairs the possibility of formation of martensite. Also, for most fluids, a phase change occurs gradually, starting at the bottom of the part and moving up to its top. As a result, different thermal changes co-occur throughout the body, and consequently, there are variations in mechanical properties^3,4^. As soon as the part does not have enough thermal energy to evaporate the refrigerant, the steam capsule is extinguished, the boiling phase begins, in which the highest cooling rates of the process occur. The boiling time is influenced by the thickness of the part, the conductivity of the material, the cooling capacity of the fluid, and the bath temperature^5^. Finally, when the body is already below the boiling temperature of the fluid, convection begins, in which heat exchanges are again minimized^2,4^.

During the microstructural transformation from austenite to martensite, there is a change from the unit cell from Face-Centered Cubic (FCC) to Body-Centered Tetragonal (BCT), increasing the volume of the unit cell. The volume expansion accompanying the martensite transformation is between 2 and 4%, depending on the chemical composition of the material^6^. This volume increase causes compressions and tensile inside the material, resulting in residual stresses after the process end. Besides, the gradual transition between the first two phases of quench hardening, steam film, and boiling causes non-uniform elevation of the crystalline network because the microstructure of the lower sample turns into martensite first than the rest, increasing the magnitude of residual stresses. When sufficiently high, the residual stresses can promote distortions and cracks in the solid, especially on long parts, due to the high cooling gradients developed during the process^4,7^.

The aqueous polymer solutions are homogeneous mixtures of polymers dissolved in water. As well as other quench hardening fluids, they also develop the three stages during treatment. However, polymers that do not present average solubility in water present a gradient of polymer concentration towards the interface between liquid and vapor. In these situations, the rupture of the steam film is sudden, causing the extraction of more uniform heat, which is followed by boiling, initiated by the base of the specimen^8,9^.

The concentration of polymers in the solution alters its heat withdrawal capacity from the sample. As a rule, the increase in concentration decreases the cooling capacity, as it increases the viscosity of the fluid, also increasing the thickness of the steam film, increasing the time required for the extinction of the vaporized layer, resulting in a slower cooling of the sample^10^. Also, polymeric solutions have additional advantages over other quench hardening fluids as they are biodegradable, non-toxic, and non-flammable^9^. One of the polymers used in the quench hardening process is Polyvinylpyrrolidone (PVP). Due to the solubility characteristics of this polymer, when it comes into contact with the sample under high temperature, the thermal energy is sufficient to separate the hydrogen solution bonds, and part of the polymer is attached to the specimen. So, the film becomes more unstable and is more quickly extinguished. When it cooled, the mixture becomes homogeneous again, but a thin layer of PVP remains attached to the sample, making it difficult to change the heat and extending the boiling and convection phases^11,12^.

In general, the thermal exchange rates of solutions composed of PVP develop intermediate rates between water and mineral oil, depending mainly on their concentration. Heat exchange rates are significantly reduced as PVP is added to the solution, approximately between 20 and 25% of maximum rates for every 5% PVP addition. However, the time for thermal equilibrium is more extended, mainly due to the increased boiling and convection duration^12–14^.

AISI 1045 is a steel with a medium amount of carbon in its composition with a limited hardening capacity compared to other medium-carbon steels with a more significant amount of alloying elements. Thus, it is expected that when quenched, the outermost layers of the material have a higher amount of martensite and develop higher hardness^1^. Due to this amount of carbon, it is common for this steel to have martensite in lath form at an average cooling speed. Besides, even if it is not in an expressive quantity, some of the austenite may be retained in the process^15^.

The cooling of this material through the use of aqueous polymeric solutions based on Polyalkylene Glycol (PAG) demonstrated considerable variation in its microhardness. While the 10% solution developed approximately 420 HV and 360 HV for the surface and core, respectively, the increase to 20% obtained 290 and 280 HV, and for 30%, it had 270 and 240 HV. Thus, we verified that the PAG increase in the solution approximates the results obtained for the quenching of these samples in oil, which resulted in indices of 260 and 240 HV^16^. The experiments carried out using PVP in the quenching of AISI 4140 steels demonstrate that the concentration of 10% obtained 98.7% of martensite on the surface of the material. With the increase in concentrations to 15, 20, and 25%, the transformation without diffusion decreased, and the indices obtained were respectively 92.3, 92.1, and 90.8%. As a result, microhardness also showed a lower magnitude according to the growth in PVP concentration. For the lowest concentration, the maximum was 910 HV, and it decreased according to the increase in polymers in the solution, measuring 874, 844, and 830 HV, respectively^17^.

This work aims to observe the consequences of the variation in the concentration of aqueous polymeric solutions based on PVP when used to cool cylindrical samples of AISI 1045 steel in the quenching. This investigation will be carried out by analyzing the microstructure, constructing the microhardness profile, and evaluating the Miller indices present in the material after performing the quenching procedure with cooling in concentrations of 10, 15, 20, and 25% of PVP.

## Material and methods

Before the execution of the heat treatment, the materials used in the study were characterized. The first step was to check the chemical composition of the steel, ensuring that it had the specified quantities. In this way, samples of the material were subjected to optical spectrometry, using the Foundry-Master Pro optical spectrometer. The results measured in the test were compared with limits established by ASM (American Society for Metals) for AISI 1045 steel.

In turn, the quench fluids were also characterized to know their dynamic viscosity, which significantly changes the cooling capacity. The polymer used was the PVP produced by the company Durferrit, model Durquench 90, and the aqueous solutions applied had concentrations of 10, 15, 20, and 25%. To measure kinematic viscosity, a Cannon–Fenske capillary viscometer, model GMBH-G65719 immersed in water at 25 °C was used. Two capillary models were applied in measuring, for concentration 10%, a number 75 was used and a number 100 to other fluids, because these had difficulties to flow in the capillary 75. The flow time in the capillary was measured, and the kinematic viscosity was calculated by the Eq. (1): ν = K.∆t, where ν represents the kinematic viscosity in centiStokes (cSt), ∆t the flow time in seconds (s) and K the constant of each capillary in mm^2^/s. The density of the solution was measured by liquid pycnometry, using a 25 ml pycnometer calibrated with water at 20 °C to observe the influence of polymer in the density of the solution and subsequent measurement of the dynamic viscosity. Finally, the dynamic viscosity was calculated from Eq. (2): μ = ρ.ν, in which μ is the dynamic viscosity in centipoise (cP) and ρ is the density of the fluid in kg/m^3^.

The steel samples were cylindrical, which were 25.4 mm in diameter and 10 mm high. In each concentration of the polymeric solution, three samples were subjected to quenching, guaranteeing the legitimacy of the study through a test and another counter-test of the results obtained. These specimens were heated until their complete austenitization in a resistive oven, model 3000, EDG brand. This stage lasted 60 min, and the heating temperature was 880 °C. The parts were cooled by immersion in a tank containing 5000 cm^3^ of PVP based solutions, which were continuously agitated by a recirculation pump with an outlet speed equal to 0.7 m/s. After this process, the samples were again placed in the oven at a temperature of 180 °C for 60 min to perform their tempering.

The materials characterization after the treatment was performed by optical microscopy, microhardness, and x-ray diffraction. The samples were sectioned in an axial direction to perform these procedures, passing the cutting plane through the center of the cylinders. The samples were then sanded using the sandpaper sequence containing 120, 240, 320, 400, 500, 600, and 1200 grains/cm^2^. After that, polishing occurred in a circular polishing machine, using aluminum oxide abrasive. In the last stage, the steel surface was then revealed with Nital 2%, with an immersion time of 8 s.

Optical microscopy aimed to evaluate the types of microstructures formed in the heat treatment and quantize them, making it possible to compare the effect of fluid concentrations. For this process, an Olympus GX 51S reflected light optical microscope was used, capturing images with 500 times magnification. The images were obtained in two different regions of the sectioned faces: close to the surface and in the core of the samples. The amount of each microconstituent in the material was calculated using the ImageJ software. The microstructures were differentiated by comparing them with the standards established by the American Society for Metals (ASM), which indicates the aspects found in the phases of each steel according to the treatment to which it was submitted. When submitted to the software, the phases are identified by color, then the percentage of each of these colors is calculated, indicating the amount of each microconstituent.

The microhardness of the material was measured on a micro-durometer model Shimadzu HMV 2 T. In each sample, eight different points were measured along the radial direction of the piece, and the points were 1.8 mm apart. The first was positioned 0.1 mm from the surface while the last to 12.7 mm, coinciding with the center of the body. Each measurement applied a 0.3 N load over 10 s. After obtaining the results, a microhardness curve of the samples was plotted, comparing the differences in each measured position and the effects of the variation in fluid concentration. Finally, x-ray diffraction was performed using a Bruker X-Ray diffractometer, model D8 Advance. This procedure was intended to get the Miller indices of the material, which allows identifying the phases present in the steel, and confirm the phases observed in optical microscopy. The spectrum was acquired between 20 and 90°, with a 0.02° pitch, using a copper anode with a wavelength of 0.15406 nm. The Diffrac EVA software was applied to identify the peaks and their respective Miller indices, enabling the interpretation of the phases.

## Results and discussion

The characterization of fluids showed the elevation of viscosity versus increased PVP. The 10% concentration of PVP using the capillary number 75 had a flow time of 371 s. Concentrations of 15, 20, and 25% have used capillary number 100, have flow times equal to 331, 454, and 664 s, respectively. Considering that the first capillary constant is equal to 0.008 and the second is 0.015 mm^2^/s, we obtained the kinematic viscosities of 2.97, 4.96, 6.81, and 9.96 cSt, increasing according to the percentage of the polymer. The densities showed no significant difference as PVP was added to the solutions. The densities obtained for 10, 15, 20, and 25% were equal to 0.9973, 0.9992, 1.0001, and 1.0012, showing a variation practically negligible. From these data, dynamic viscosity was calculated. The results of this characterization are shown in Table 1.

**Table 1:** Density, kinematic viscosity, and dynamic viscosity of the PVP polymer solutions in different measured concentrations.

Concentration	Density (kg/m^3^)	Kinematic viscosity (cSt)	Dynamic viscosity (cP)
10% PVP	0.9973	2.968	2.9601
15% PVP	0.9992	4.965	4.9612
20% PVP	1.0001	6.810	6.8105
25% PVP	1.0012	9.960	9.9720

The investigated fluid data confirm that the addition of polymer in the solution produces an increase in viscosities. This characteristic will affect the cooling capacity of the material since fluids with high viscosities develop thick and stable vapor layers, increasing the time of the first treatment phase and minimizing the capacity for martensite formation^9,11^.It is essential to consider that the kinematic and dynamic viscosities practically did not develop a significant difference due to the density values. First, there was no significant difference between the density of different fluids. Furthermore, the values were concentrated in variations very close to 1 kg/m^3^, which does not develop value in the multiplication used to obtain the kinematic viscosity. Another relevant aspect is that the viscosity values are not stable and tend to decrease as the polymeric solution is used for cooling. Thus, viscosities decrease with each quenching cycle, increasing the cooling capacity of the fluid^18^.

The analysis of AISI 1045 steel by optical spectrometry measured the number of chemical elements in the material. The observed results are shown in Table 2, together with the limits established by the ASM.

**Table 2:** Percentage of the chemical composition of AISI 1045 steel measured by optical spectrometry and limits established for the material.

Element	Carbon	Manganese	Phosphorus	Sulfur
Measured (%)	0.484	0.633	0.0077	0.015
Limits (%)	0.43–0.50	0.60–0.90	0.04 max	0.05 max

According to Table 2, the amount of carbon and residual elements is within the established limits. Besides, there is approximately 98.86% iron in the material. Thus, we can consider that the temperability of the steel will follow the expected, allowing the application in the study and comparison with other results^1,19^.

**Figure 1:**
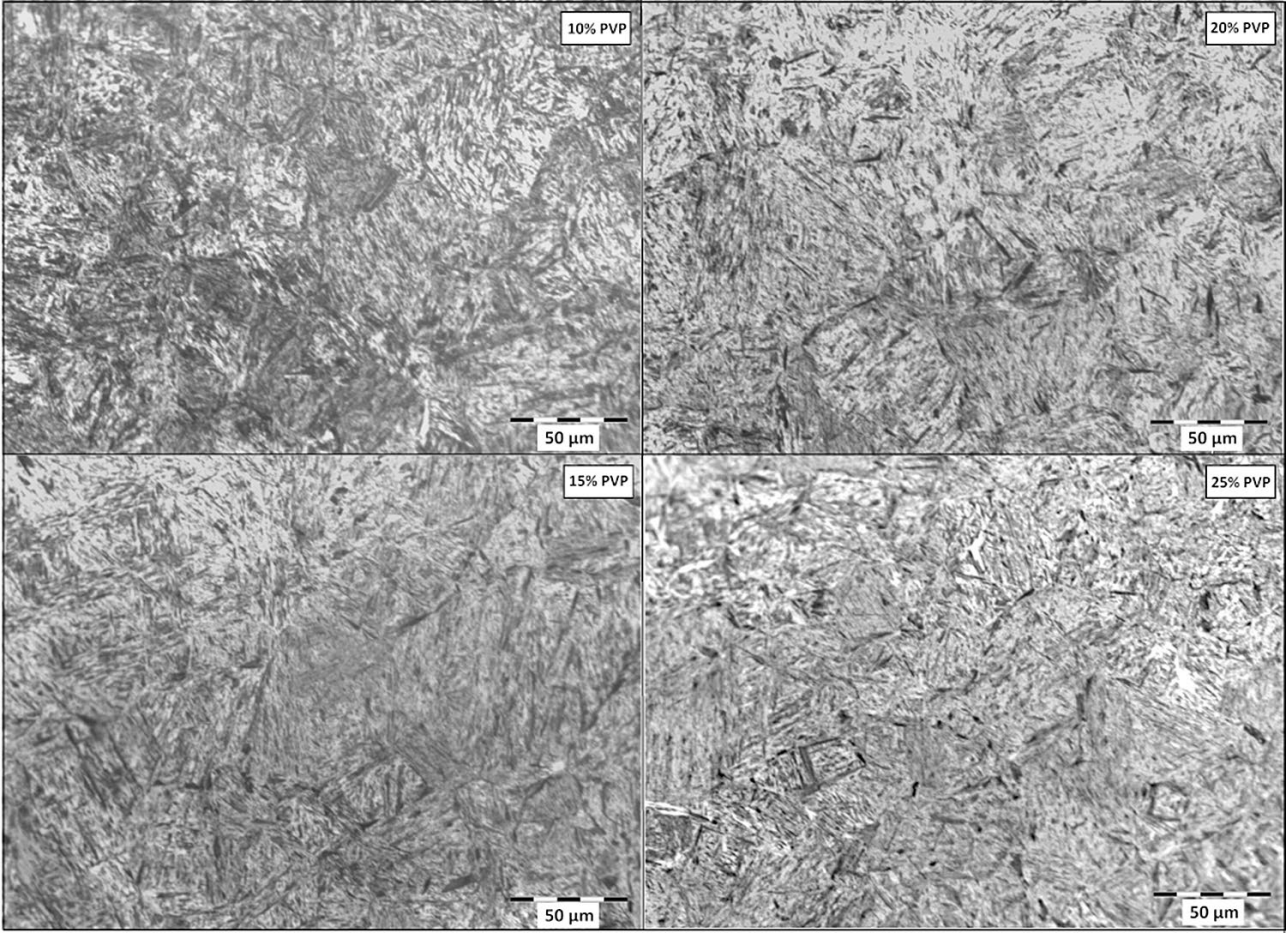
Metallography of the surface region of the quenched samples at different concentrations of PVP.

**Figure 2:**
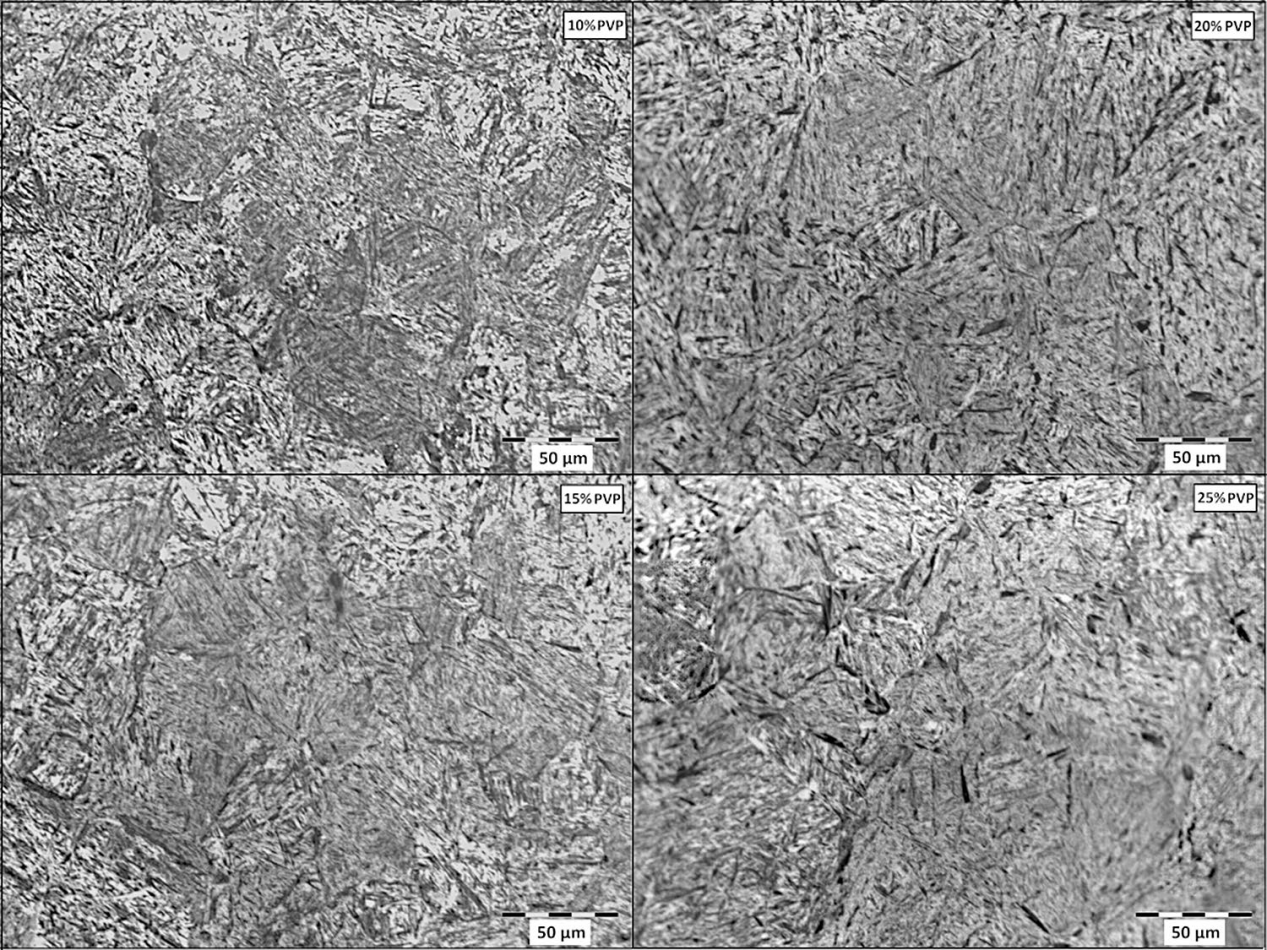
Metallography of the core region of the quenched samples at different concentrations of PVP.

Figure 1 shows the microstructure of the region near the surface of the quenched samples at the different concentrations of PVP, while Fig. 2 demonstrates the metallography obtained from the core. As the heat exchange between core and surface occurs exclusively by conduction, it is expected that the outermost layers of the material will develop more intense cooling; consequently, obtain a more significant amount of martensite. Thus, for each quench fluid, images of the core and surface of the samples were compared to observe the different characteristics of the two regions.

The observation of the micrographs shows that the quenching treatment was successful in all concentrations of PVP applied because there is a predominance of martensite, which forms the matrix of all samples. Another important observation was the existence of dispersed bainite in the material matrix. The amount of this microstructure was intensified as the concentration of the solution was increased, proving that the elevation of PVP causes loss of cooling capacity, which allows the formation of microstructures with carbon diffusion^2,10^. Moreover, the samples cooled in 20, and 25% PVP was found a small percentage of ferrite. In the material immersed in the 20% solution, this phase was observed only in the core, while for 25%, it was also observed on the surface. Ramesh and Prabuh (2016) used aqueous polymeric solutions based on PAG in various concentrations to quench AISI 1040 steel, which has a composition very similar to that applied to the present work.

**Figure 3:**
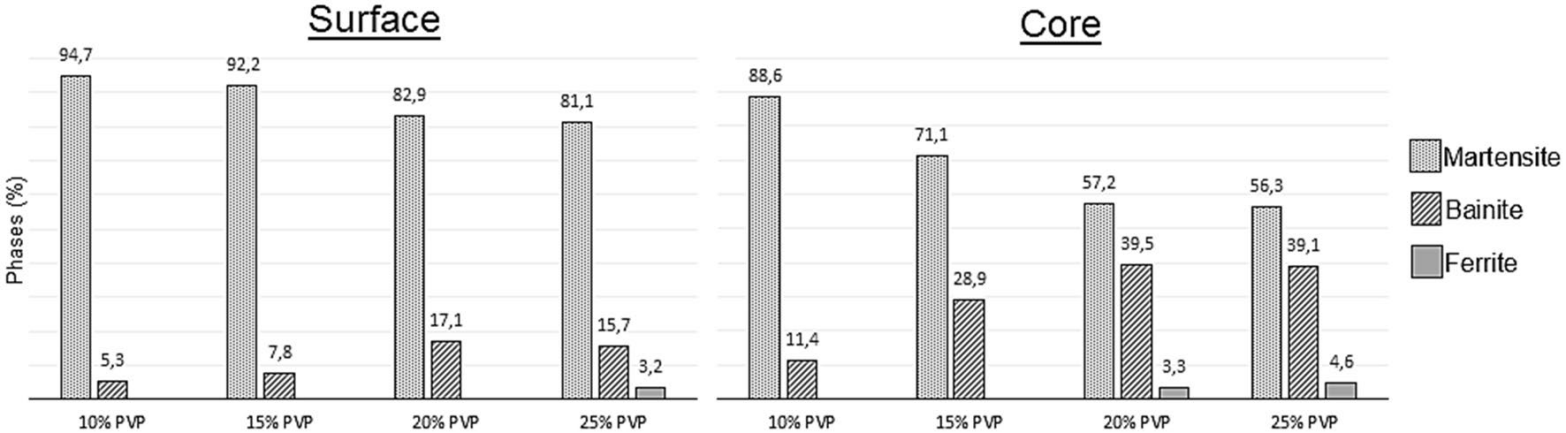
Percentage of phases resulting from cooling in different concentrations on surface and core.

Comparatively, the behavior of both results was similar. Thus, the same phases in the microstructure were formed for all polymer percentages, that is, a martensitic matrix with dispersed bainite. Also, in some cases, a small percentage of ferrite has been found and in no situation has been found retained austenite^10^. Figure 3 shows the quantification of the microstructures on the surface and in the core of samples quenched in different concentrations of PVP.

The maximum amount of martensite obtained was 94.7%, found on the sample surface cooled in 10% PVP, in the most intense heat exchange situation. On the surfaces of the bodies, the amounts of martensite are reduced as the concentration increases. Thus, cooling the material in 15, 20, and 25% of PVP, we obtained 92.2, 82.9, and 81.1% of martensite. In contrast, bainite, which is present in all quenching situations, tends to exhibit the opposite behavior. The percentages of the surfaces are increased according to the concentration, showing in the increasing order of concentration the quantities of 5.3, 7.8, 17.1, and 15.7%. It is essential to highlight a reduction in the percentage of bainite between 20 and 25% of PVP, which was contrary to expectations. Due to the slower cooling, part of the austenite that would be transformed into bainite was transformed into ferrite, which represented 3.2% of the body.

In the core of the material, similar behavior can be observed with a greater number of phases with diffusion due to less heat exchange with the fluidic medium. In these situations, at concentrations of 10, 15, 20, and 25% PVP was found respectively 88.6, 71.1, 57.2, and 56.3% of martensite. The percentage of bainite was 11.4, 27.7, 39.4, and 39.1%. There was a small reduction in bainite between 20 and 25% of PVP as with the surface. In these last two concentrations, ferrite was formed, in amounts equal to 3.3 and 4.6%, respectively. In the experiments carried out by Ramesh and Prabuh (2016), in AISI 1040 steels there was a presence of 2% ferrite at a concentration of 10% polymer. When the amount of polymer was increased to 25%, the ferrite amount also increased, developing 5%^3,10^.

There is a considerable difference in the amount of microstructure without diffusion in the different concentrations used. In addition to a gradual reduction in martensite as the PVP increases, a disparity in their percentages between the center of the samples and their outer layers can also be seen. This disparity is smaller at lower concentrations and tends to stabilize as PVP is added to the solutions. At concentrations of 10%, the martensite disparity is 6.1%. At concentrations of 10%, the disparity in martensite is 6.1%. In terms of PVP amounts of 15, 20, and 25%, we have higher and closer percentages: 21.1, 25.7, and 24.8%, respectively. The results demonstrated by Vieira et al. (2019), who carried out experiments on AISI 4140 steel, demonstrated similar behavior in 10 and 25% of PVP concentrations. In this work, the difference in the amount of martensite between the surface and the nucleus increased according to the growth of PVP concentrations. Comparing the study results, we see that the difference in 10% was very close, measured at 6.2%. The concentration of 25% had a 23.3% discrepancy, a similar index. However, because of the better hardenability of AISI 4140 steel compared to 1045, the intermediate polymer solutions developed minor differences. For 10 and 15% of concentration the inequalities were 10.8 and 13.0%^2,17^.

**Figure 4:**
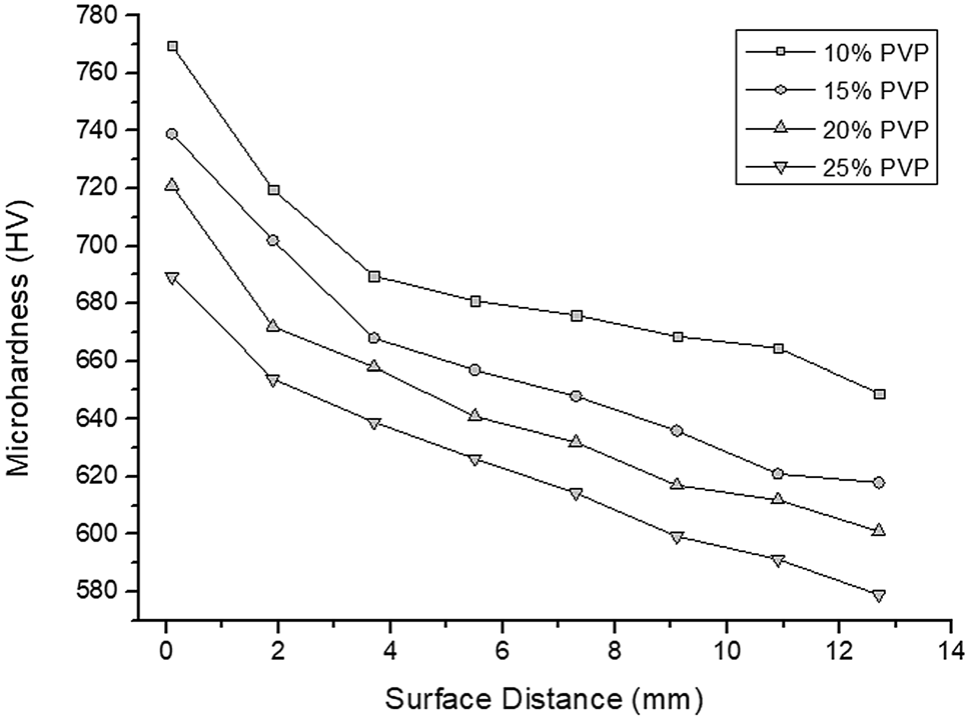
Microhardness profiles resulting from tempering at different concentrations of PVP.

The Vickers microhardness of the material, measured in the radial direction of the samples from the surface to the core, was used to build a profile comparing the magnitudes. These profiles, represented in Fig. 4, demonstrate the behavior of the material's microhardness in different cooling situations.

The profiles show two situations that cause a decrease in the hardness of the steel: the elevation of PVP in the solution and the core approach. This is because both circumstances cause the minimization of heat exchange. The first promotes an increase in fluid viscosity with the addition of PVP, making the vapor layer more stable. Due to the greater distance between the measured point and the contact surface with the fluid, the second maximizes the heat exchange by conduction, which develops a lower cooling rate^14,20^. Therefore, the cases in which the cooling is more aggressive cause higher levels of hardness, which vary from 770 to 579 HV, representing a difference of 191 HV between the most extreme situations. There is clear evidence of a decrease in microhardness since 10 and 15% of PVP concentrations develop a more intense reduction until the point of 3.7 mm. In samples of 20 and 25%, this sharp drop goes only up to 1.9 mm.

There is a correlation between the quantities of the phases present in the material and their hardness. Nunura, dos Santos and Spim (2015), demonstrated that in AISI 1045 steel, 87.6% of martensite and 12.4% of bainite develop approximately 730 HV^21^. Thus, it can be seen that the microhardness results are consistent with the microstructures observed in the material, triggering greater hardness in regions with a more significant amount of martensite.

Gao et al. (2014) found approximately 700 HV of hardness on the surface of AISI 1045 steel samples heated by induction and cooled in water when measured at 1.9 mm from the surface; the microhardness was reduced to values close to 460 HV^22^. Another study using 10 and 20% PAG solutions in AISI 1045 steel quenching measured lower microhardness levels. At positions close to 2 mm from the surface, approximately 430 HV was found for 10% concentration and 300 HV in solution with 20%^16^.

Ramesh and Prabhu (2016) estimated that an AISI 1040 steel with a 10% PAG concentration develops a microhardness of 565 HV and increases the polymer to 25%, the index approached 422 HV^10^. These divergences occur not only due to the use of a different polymer but also due to the lack of agitation in the compared studies. Regarding the type of polymer, it is necessary to consider that the heat removal capacity of PVP is greater than that of PAG^23^. Furthermore, agitation increases the heat exchange capacity of fluids. Consequently, there is a considerable improvement in the martensitic transformation and an increase in the hardness of the cooled materials^24^.

Observing the results of phase quantification and microhardness, we can associate the effect of increased viscosity with variations in thermal energy exchange intensity. Thus, as observed in the first results, the solutions containing lower percentages of PVP develop more martensite and, consequently, microhardness. To ensure this, we can observe that the fluid with a lower dynamic viscosity, equal to 2.9601 cP, produced steels with the highest amount of martensite and the most significant microhardness of this work, which are respectively 94,7% and 770 HV, measured on the surface of the sample. As the dynamic viscosity was increased, the stability of the vapor film was also increased. Thus, making it more difficult to break the steam film and prolong the time for this to occur. Thus, the amount of martensite and hardness were minimized as the dynamic viscosity was increased. Therefore, considering the sample surfaces, for dynamic viscosities equal to 4.9612, 6.8105, and 9.972, the percentages of 92.2, 82.9, and 81.1% of martensite and microhardnesses of 721, 711, and 689 HV were measured respectively^9,11^.

**Figure 5:**
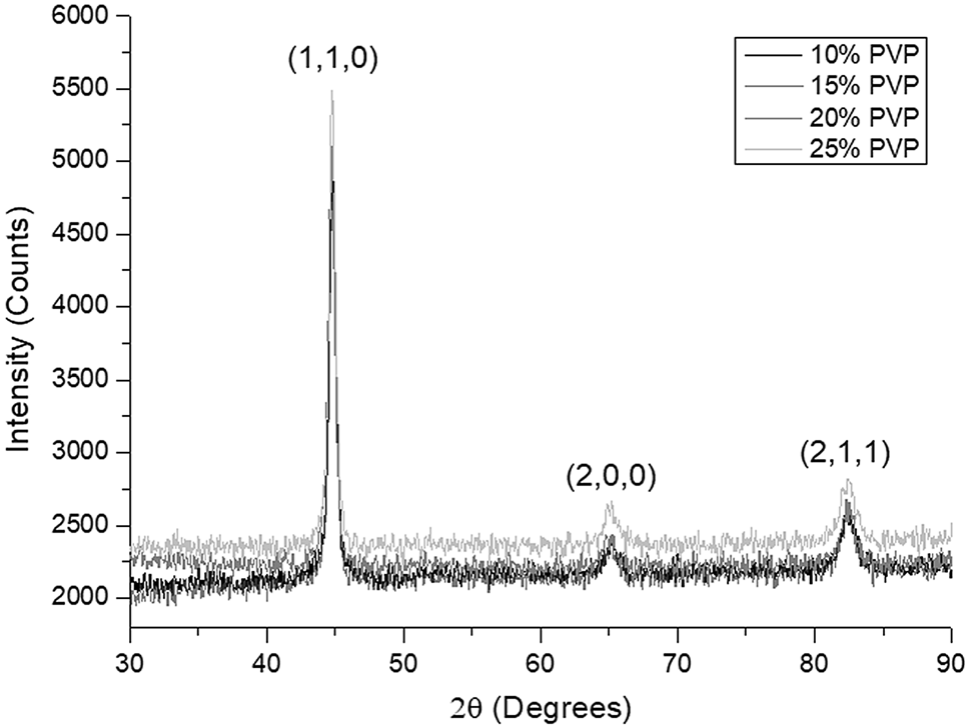
X-ray diffraction spectra of samples cooled at different concentrations of PVP.

The spectra generated by Diffrac EVA software provided the opportunity to identify the peaks and their Miller indices. Thus, allowing the observation of the plans and the association with their microstructures. The results of these analyzes are shown in Fig. 5.

The spectral patterns did not show a considerable divergence between the PVP solutions in which the material was cooled. Therefore, we verified that the existing phases for the different fluidic media used are the same, varying only the quantities. We can see three well-defined peaks in the results, which differ in intensity and angle of incidence. The first and the second peak indicate the Miller indices of the planes (110) and (211) and are located at 2θ = 44,4° and 2θ = 82°. These plans refer to martensite. The lowest intensity peak, positioned at 2θ = 64.7°, is relative to alpha iron, present in both bainite and ferrite. It is essential to say that (200) is also one of the gamma iron indices. However, when this component is present, the peak is positioned at 2θ ≈ 51°^25,26^.O Other indexes referring to austenite are: (111), (200), (220), (311), and their multiples, which were not observed in any of the spectra. Thus, we confirm that there was no formation of austenite retained during the cooling of the steel. Therefore, we affirm that the x-ray diffraction results are also consistent with the microstructures observed in the material's microscopy^27^.

## Conclusions

1. PVP in concentrations between 10 and 25% can be used as a quenching fluid for AISI 1045 steel because it is successful in treatment considering that it produces martensitic matrix in the material. Besides, the formation of martensite decreases as the PVP concentration is high.
2. In all concentrations applied, there is the formation of phases with carbon diffusion. Bainite is present in all polymeric liquid concentrations used, and ferrite appears as from 20% PVP concentrations.
3. The microhardness of the samples shows a reduction as the concentration increases, demonstrating considerable variation between surface and core of the material. This behavior is associated with the amount of martensite at different concentrations and distance from the sample surface.
4. There was no formation of retained austenite in any cooling situation.
5. The addition of PVP to the aqueous solution has no significant effect on density, but it increases the kinematic and dynamic viscosities, decreasing the cooling capacity of the fluids.
